# Hospital Financial Structure and Unplanned Rehospitalization Following Acute Myocardial Infarction in the United States

**DOI:** 10.1016/j.jacadv.2026.103218

**Published:** 2026-09-10

**Authors:** Preston Leung, Jaden Rahmani, Owen Lai, Giselle Porter, Isabella Chilian, Radoslav Zinoviev, Peyman Benharash

**Affiliations:** a Center for Advanced Surgical and Interventional Technology, Department of Surgery, David Geffen School of Medicine at UCLA, Los Angeles, California, USA; b Department of Surgery, David Geffen School of Medicine at UCLA, Los Angeles, California, USA; c Department of Cardiology, David Geffen School of Medicine at UCLA, Los Angeles, California, USA

**Keywords:** acute myocardial infarction, health disparities, hospital ownership, Hospital Readmissions Reduction Program, outcomes, rehospitalization

## Abstract

**Background:**

Acute myocardial infarction (AMI) affects ∼805,000 individuals in the United States annually and remains a leading cause of rehospitalization. The Hospital Readmissions Reduction Program established 30-day readmissions as a quality benchmark for AMI care. Although for-profit hospitals have shown higher readmission rates than not-for-profit counterparts, ownership-related trends in readmissions and outcomes following AMI remain poorly characterized.

**Objectives:**

We examined 30-day nonelective readmission rates and clinical outcomes in readmitted AMI patients stratified by hospital ownership from 2013 to 2022.

**Methods:**

Adult nonelective AMI hospitalizations were identified from the 2013 to 2022 Nationwide Readmissions Database and stratified by hospital ownership. Temporal trends were assessed with Cuzick nonparametric test. Among readmitted patients, outcomes were compared using doubly robust methodology.

**Results:**

Of the 4,636,970 AMI admissions, 534,576 (11.5%) had an unplanned 30-day readmission. Of the 399,800 patients readmitted to the index hospital, 66,394 (16.6%) were treated at for-profit facilities. Over the study period, readmission rates declined at not-for-profit hospitals (11.5% to 10.5%; *P* < 0.001) but remained stable at for-profit hospitals (13.0% to 12.7%; *P* = 0.09). Rate reductions at for-profit hospitals were limited to Medicare beneficiaries and coronary artery bypass grafting patients. Following risk adjustment, for-profit status was independently associated with greater odds of 30-day nonelective readmission (adjusted OR [AOR]: 1.15), respiratory (AOR 1.11) and renal complications (AOR 1.04) at readmission, and nonhome discharge (AOR: 1.07).

**Conclusions:**

For-profit status was associated with higher 30-day nonelective readmission rates and greater odds of renal and respiratory complications at readmission and nonhome discharge. These findings may inform policies addressing ownership-related disparities in AMI care.

Acute myocardial infarction (AMI) is a major life-threatening event, affecting approximately 805,000 individuals in the United States each year and conferring a significant economic burden.^1^^,^^2^ Despite advances in early diagnosis and revascularization, patients hospitalized with AMI remain at risk for early rehospitalization, with nearly two-thirds occurring within the first 2 weeks after discharge.^3^ With evidence suggesting that a proportion of these readmissions are preventable, rehospitalization following AMI has become an important quality metric and concern in health policy. Implemented in 2012, the Hospital Readmissions Reduction Program (HRRP) was introduced to penalize hospitals with higher than expected 30-day readmission rates for conditions such as AMI, heart failure, and coronary artery bypass grafting (CABG).^4^^,^^5^

Although a substantial body of literature has examined center-level variation in readmission rates following AMI, the relationship between hospital ownership and readmission quality metrics remains less defined.^6^^,^^7^ Prior work has identified ownership-associated differences in AMI care patterns, including variation in adverse event rates and procedural utilization across hospital financial structures.^8^^,^^9^ Mittal et al^10^ found that for-profit hospitals had higher readmission rates following AMI and other HRRP-targeted conditions, yet noted that the mechanisms for these differences are not well understood. Despite growing evidence, important questions persist regarding how hospital ownership influences 30-day nonelective readmissions and clinical outcomes following AMI in the HRRP era.

To address this gap, the present study examined the association between hospital for-profit status and temporal trends in 30-day nonelective readmission rates and clinical outcomes in a national cohort of patients with AMI. We hypothesized that hospital ownership type would be associated with differential trends in 30-day nonelective readmission rates. We further hypothesized that among readmitted patients, for-profit status would be associated with greater rates of readmission-related complications.

## Methods

**Figure 1:**
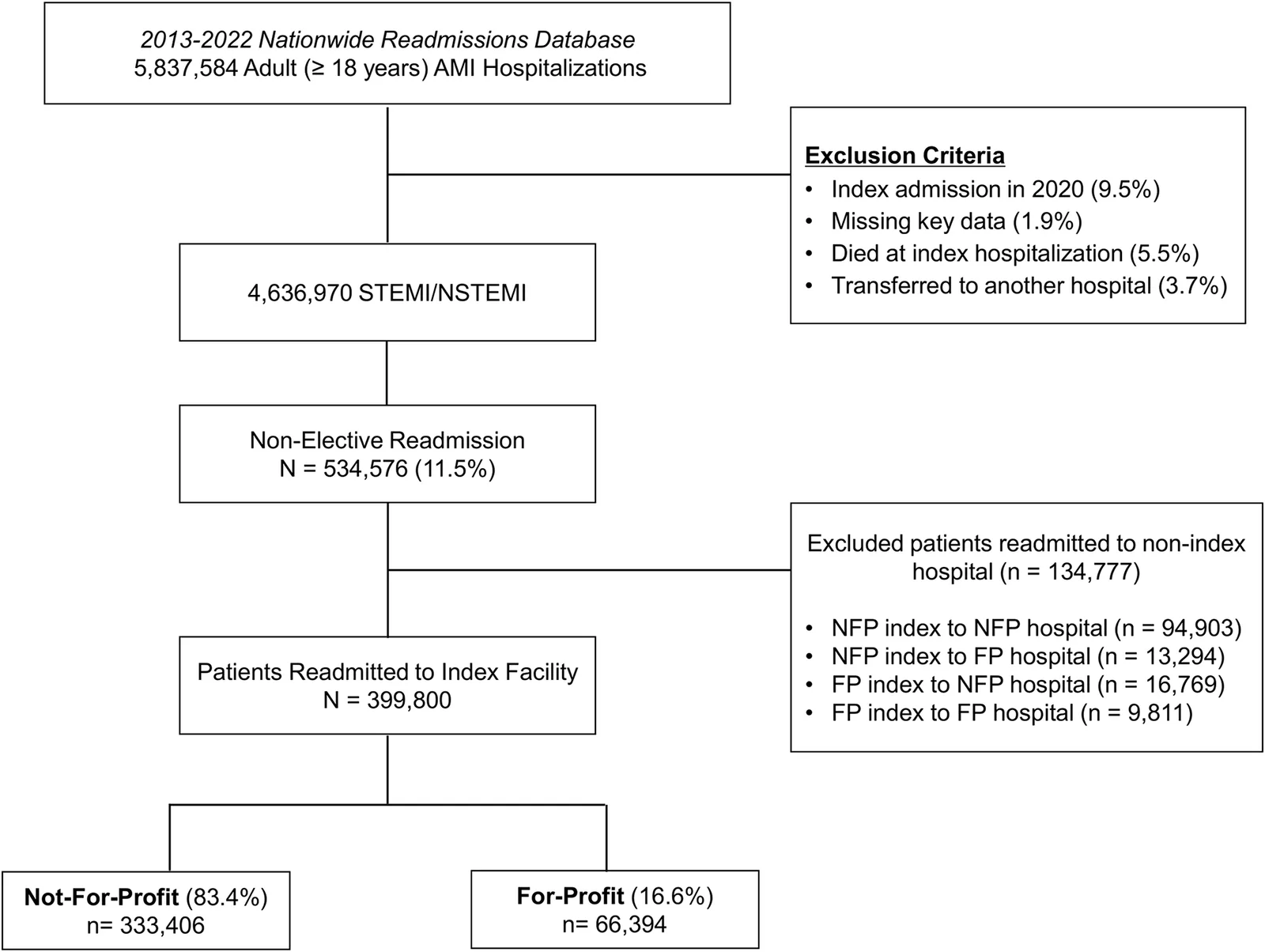
Study CONSORT Diagram of Survey-Weighted Estimates Of the 4,636,970 records included for temporal trends analysis, 534,576 patients readmitted nonelectively within 30 days. Of these readmitted patients, 399,800 (74.8%) were readmitted to the index facility and 134,777 (25.2%) were readmitted to a different hospital. AMI = Acute myocardial infarction; FP = for-profit; NFP = not-for-profit; NSTEMI = non–ST-elevation myocardial infarction; STEMI = ST-segment elevation myocardial infarction.

All adult (≥18 years) nonelective hospitalizations for AMI were abstracted from the 2013 to 2022 Nationwide Readmissions Database (NRD) using previously validated International Classification of Disease-9th Revision (ICD-9) and *-*10th Revision (ICD-10) diagnosis codes. Both ST-segment elevation myocardial infarction (STEMI) and non-STEMI diagnosis codes were used to identify AMI hospitalizations as reported elsewhere (Supplemental Table 1).^3^^,^^11^ In addition, AMI patients undergoing operative management, including percutaneous coronary intervention (PCI) and CABG, were captured through ICD-9/-10 procedure codes (Supplemental Table 1). Maintained by the Agency for Healthcare Research and Quality, NRD is the largest national readmission database in the United States and provides accurate national estimates for ∼60% of annual hospitalizations.^12^ Readmissions are tracked using unique patient identifiers across hospitals within each state and calendar year. For the temporal trend analysis, all eligible AMI admissions were retained regardless of readmission status. For the outcome analysis, the analytic cohort was restricted to patients who were nonelectively readmitted to the index hospital within 30 days. Patients requiring unplanned readmission within 30 days of index discharge at a for-profit hospital comprised the *for-profit* cohort, whereas others treated at private nonprofit or government-owned hospitals, comprised the *not-for-profit* group. Records entailing in-hospital mortality, transfer to another hospital, or missing key data on age, sex, cost, mortality, payer, or income were excluded (Figure 1). Patients admitted during calendar year 2020 were excluded to avoid confounding from the COVID-19 pandemic and ensure comparisons under standard hospital operations.^13^ Those readmitted to a nonindex hospital were excluded from the outcomes analysis to permit attribution of complications and resource use to the same facility. Lastly, the index admission mortality comparison was performed in 4,958,825 admissions meeting all exclusion criteria except in-hospital death, because survival to discharge is a precondition for inclusion in the primary readmission cohort used elsewhere in this study.

Patient, procedure, and hospital characteristics were defined according to the Healthcare Cost and Utilization Project data dictionary.^14^ The van Walraven modification of the Elixhauser Comorbidity Index was used to quantify the burden of chronic conditions.^15^ Previously validated *ICD-9/10* codes were used to identify relevant comorbidities and complications (Supplemental Table 1).^3^^,^^11^ Complications during readmission included cardiac (cardiac arrest, ventricular fibrillation, ventricular tachycardia, and tamponade), infectious (surgical site infection, sepsis), renal (acute kidney injury), and respiratory (pneumonia, pneumothorax, acute respiratory distress syndrome, respiratory failure) complications, as well as the need for blood transfusion.^16^ Episodic costs were calculated by applying center-specific cost-to-charge ratios to overall charges and adjusted for inflation in accordance with the 2022 Personal Healthcare Price Index.^12^

The primary objective was to assess the temporal trends in 30-day nonelective readmission rates following AMI from 2013 to 2022, stratified by hospital ownership. Among patients with 30-day readmission, secondary outcomes included readmission-related complications, in-hospital mortality, and hospital resource utilization at readmission.

Categorical variables are reported as group proportions (%), and continuous variables as medians with IQR. Given the large sample size, the significance of intergroup differences at the patient and hospital levels was evaluated utilizing standardized mean difference (|SMD|) with the absolute value ≥0.1 considered significant. Pearson chi-square test or Mann-Whitney *U* test were also used to compare groups, as appropriate. Temporal trends were evaluated using Cuzick nonparametric test for trend (nptrend). A doubly robust risk adjustment strategy was developed with entropy balancing as the first stage to balance the comparison cohort with respect to covariate distribution.^17^ Multivariable regression models were then developed to determine the independent association of hospital for-profit status with readmission rates and outcomes of interest. To reduce risk of overfitting and enhance generalization, covariate selection was guided by elastic net regularization. Models were optimized by evaluating receiver operating characteristics (C-statistics), calibration plots, and coefficients of determination (R^2^), as appropriate. Regression estimates are reported as adjusted ORs (AORs) for logistic and β-coefficients for linear models. The cumulative hazards of 30-day nonelective readmission were examined using a Nelson-Aalen methodology between *for-profit* and *not-for-profit*. Temporal trend analyses were performed in the full index cohort and outcomes analyses were restricted to patients readmitted to the index hospital. A Fine-Gray subdistribution hazard model was used to assess whether in-hospital mortality at readmission influenced the primary readmission finding.^18^ E-values were computed to assess the robustness of primary outcome associations to potential unmeasured confounding.^19^ ORs were converted to approximate risk ratios using outcome-specific baseline prevalences from the not-for-profit reference group. Statistical significance was set at α = 0.05.

All statistical analyses were performed using Stata 18.0 (Stata Corp LLC). Due to the deidentified nature of the NRD, this study was deemed exempt from full review by the Institutional Review Board at the University of California-Los Angeles.

## Results

**Figure 2:**
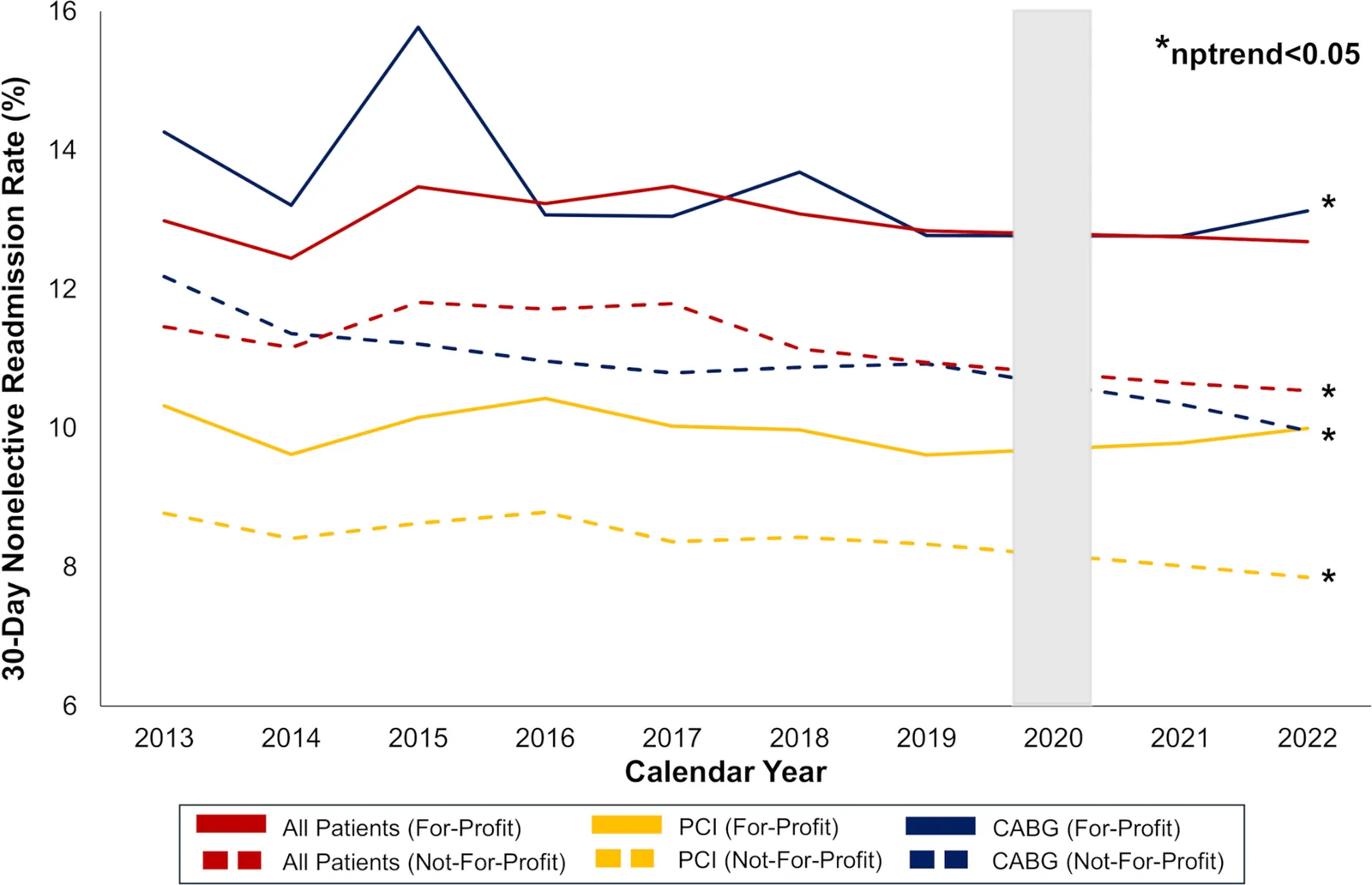
30-Day Acute Myocardial Infarction Readmission Trends by Ownership and Procedure Type Temporal trends in survey-weighted readmission rates were evaluated using Cuzick nonparametric test for trend, with 2020 excluded to avoid confounding from the COVID-19 pandemic. Readmission rates at for-profit hospitals (solid lines) showed a statistically significant decline in CABG readmissions (*P* = 0.01). Not-for-profit hospitals (dotted lines) displayed a significant decrease over the years in all patients, including those receiving PCI and CABG. Gray shading indicates 2020 exclusion. ∗Statistical significance, nptrend <0.05. CABG = coronary artery bypass grafting; nptrend = nonparametric trend; PCI = percutaneous coronary intervention.

**Figure 3:**
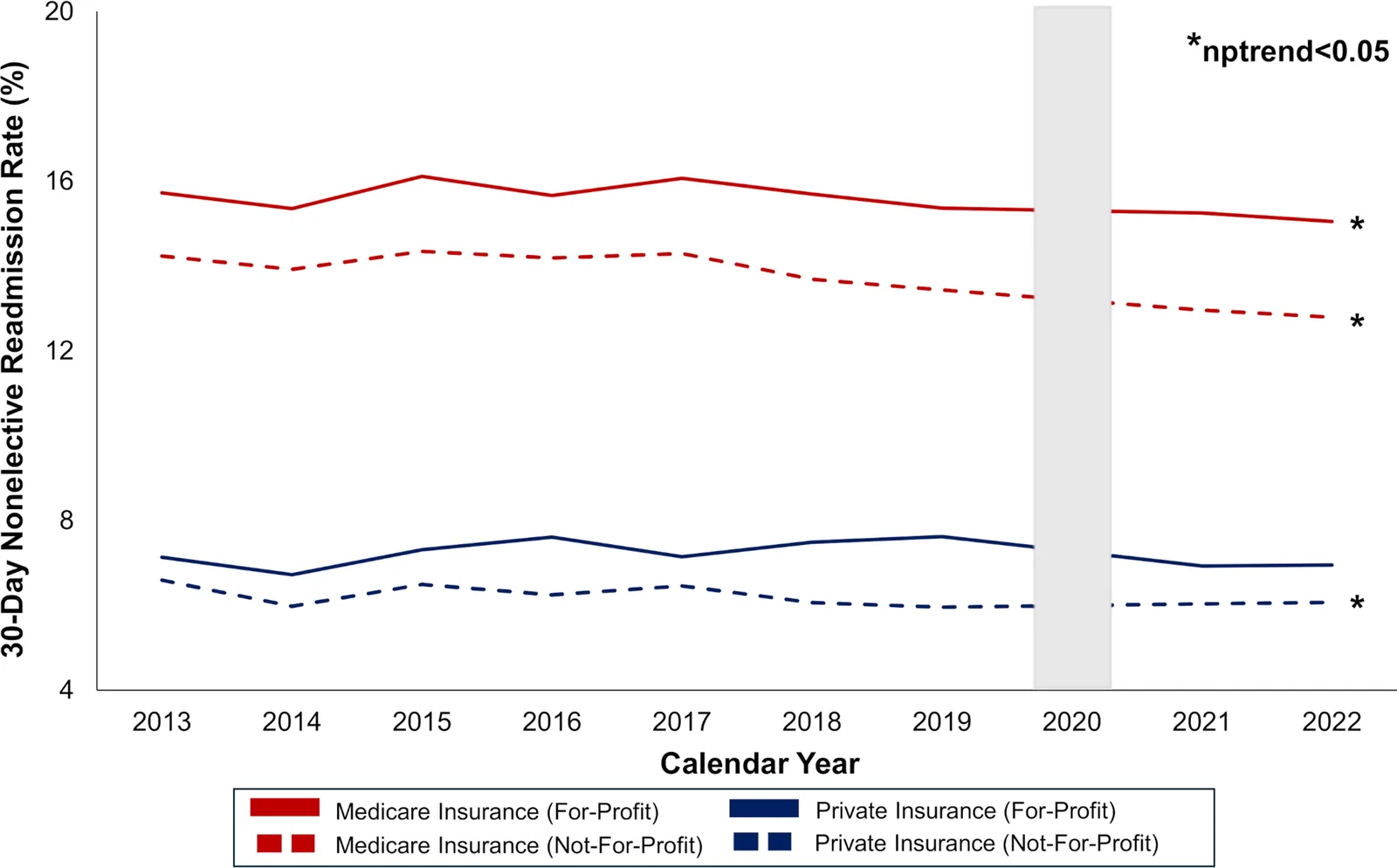
30-Day Acute Myocardial Infarction Readmission Trends by Ownership and Insurance Status Temporal trends in survey-weighted readmission rates were evaluated using Cuzick nonparametric test for trend, with 2020 excluded to avoid confounding from the COVID-19 pandemic. For-profit hospitals (solid lines) exhibited a significant reduction in readmission rates among patients with Medicare insurance. Not-for-profit hospitals (dotted lines) a displayed significant decrease over the years in patients with private or Medicare insurance. Gray shading indicates 2020 exclusion. ∗Statistical significance, nptrend <0.05. Abbreviation as in Figure 2.

Of an estimated 4,636,970 AMI admissions (27.5% STEMI), 534,576 (11.5%) were nonelectively readmitted within 30 days (Figure 1). Readmission rates at not-for-profit hospitals declined from 11.5% in 2013 to 10.5% in 2022 (*P* < 0.001) (Figure 2). The trend across the same years at for-profit facilities remained steady (2013: 13.0% vs 2022: 12.7%; *P* = 0.09). This pattern was consistent among patients undergoing PCI, with declining readmission rates at not-for-profit hospitals (2013: 8.8% vs 2022: 7.9%; *P* < 0.001) and stable rates at for-profit hospitals (2013: 10.3% vs 2022: 10.0%; *P* = 0.10). Among CABG recipients, however, readmissions decreased at both not-for-profit (2013: 12.2% vs 2022: 10.0%; *P* < 0.001) and for-profit institutions (2013: 14.3% vs 2022: 13.1%; *P* = 0.01) (Figure 2). Stratified by insurance status, privately insured patients experienced declining readmission rates at not-for-profit centers (2013: 6.6% vs 2022: 6.1%; *P* < 0.001) (Figure 3), whereas rates at for-profit centers remained steady (2013: 7.1% vs 2022: 6.9%; *P* = 0.88). In contrast, readmissions among Medicare beneficiaries decreased at both not-for-profit (2013: 14.2% vs 2022: 12.8%; *P* < 0.001) and for-profit hospitals (2013: 15.7% vs 2022: 15.0%; *P* = 0.005).

Approximately 399,800 patients were nonelectively readmitted to the index hospital within 30 days, with 66,394 (16.6%) rehospitalized at for-profit facilities (Figure 1). All subsequent analyses were conducted among patients readmitted to the index hospital. Relevant patient and hospital characteristics are shown in Table 1. Patients treated at for-profit centers were more often from the lowest income quartile (41.7% vs 30.8%; SMD = 0.34). For-profit patients were more often treated at metropolitan nonteaching centers (40.8% vs 22.3%; SMD = 0.30) and hospitals with lower annual AMI caseload (129 [47-248] vs 148 [39-293]; SMD = 0.17). At baseline, for-profit patients faced similar rates of in-hospital mortality at readmission (5.4% vs 5.8%; SMD = 0.02). Rates of cardiac (5.5 vs 6.5; SMD = 0.04), respiratory (17.7 vs 15.4; SMD = 0.07), and renal complications (25.3 vs 25.3; SMD <0.01) were comparable between the groups. For-profit patients incurred reduced hospitalization costs at both index admission ($17,000 [$10,000-$28,800] vs $21,000 [$12,100-$35,700]; SMD = 0.21) and readmission ($7,800 [$4,500-$14,900] vs $10,100 [$5,800-$19,400], SMD = 0.20) (Table 2).

**Table 1:** Demographic, Clinical, and Hospital Characteristics of AMI Patients With a 30-Day Nonelective Readmission, Stratified by For-Profit Status

	Not-For-Profit (n = 333,406)	For-Profit (n = 66,394)	|SMD|
Age, y, median [IQR]	71 [61-81]	71 [61-81]	0.01
Female (%)	43.8	44.2	<0.01
Elixhauser Comorbidity Index, median [IQR]	4 [3-6]	4 [3-5]	0.05
Operative type			0.02
Percutaneous coronary intervention	32.6	32.7	
Coronary artery bypass grafting	8.7	9.1	
STEMI (%)	21.5	19.8	0.03
Income quartile (%)			0.34
>75%	17.5	9.2	
51%-75%	23.9	18.8	
26%-50%	27.8	30.3	
0%-25%	30.8	41.7	
Payer (%)			0.06
Private	14.1	12.7	
Medicare	71.1	71.2	
Medicaid	9.5	9.2	
Self/other	5.4	6.9	
Comorbidities (%)			
Anemia	4.2	4.0	0.01
Cardiac arrhythmias	39.0	36.5	0.06
Chronic kidney disease	9.9	8.4	0.05
Chronic pulmonary disease	30.9	33.6	0.07
Congestive heart failure	55.7	53.9	0.04
Diabetes	43.7	42.7	0.03
Hypertension	58.4	59.1	0.01
Liver disease	4.0	3.5	0.03
Obesity	14.5	14.0	0.02
Peripheral vascular disease	12.1	11.8	0.02
Hospital bed size (%)			0.34
Large	63.7	43.0	
Medium	24.5	41.0	
Small	11.9	16.0	
Hospital teaching status (%)			0.30
Non-metropolitan	7.1	6.4	
Metropolitan nonteaching	22.3	40.8	
Metropolitan teaching	70.6	52.8	
Annual hospital volume, median [IQR]	148 [39-293]	129 [47-248]	0.17

STEMI = ST-segment elevation myocardial infarction; SMD = standardized mean difference.

**Table 2:** Unadjusted Outcomes of AMI Patients With 30-Day Nonelective Readmission, Stratified by For-Profit Status

	Not-For-Profit (n = 333,406)	For-Profit (n = 66,394)	|SMD|
Clinical outcomes			
Mortality during readmission (%)	5.8	5.4	0.02
Complications during readmission (%)			
Cardiac	6.5	5.5	0.04
Respiratory	15.4	17.7	0.07
Infectious	9.7	10.1	0.02
Renal	25.3	25.3	<0.01
Blood transfusion	9.7	8.4	0.06
Any	45.2	45.7	0.01
Resource utilization			
Hospitalization cost, $1000s, median [IQR]	21.0 [12.1-35.7]	17.0 [10.0-28.8]	0.21
Readmission costs, $1000s, median [IQR]	10.1 [5.8-19.4]	7.8 [4.5-14.9]	0.20
Length of stay, days, median [IQR]	4 [3-8]	4 [3-8]	0.03
Nonhome discharge (%)	23.3	24.4	0.03

Abbreviation as in Table 1.

Index admission in-hospital mortality was lower at for-profit compared with not-for-profit hospitals (6.26% vs 6.53%; *P* < 0.001). Mortality at both for-profit (*P* = 0.97) and not-for-profit (*P* = 0.77) hospitals remained stable over the study period.

**Figure 4:**
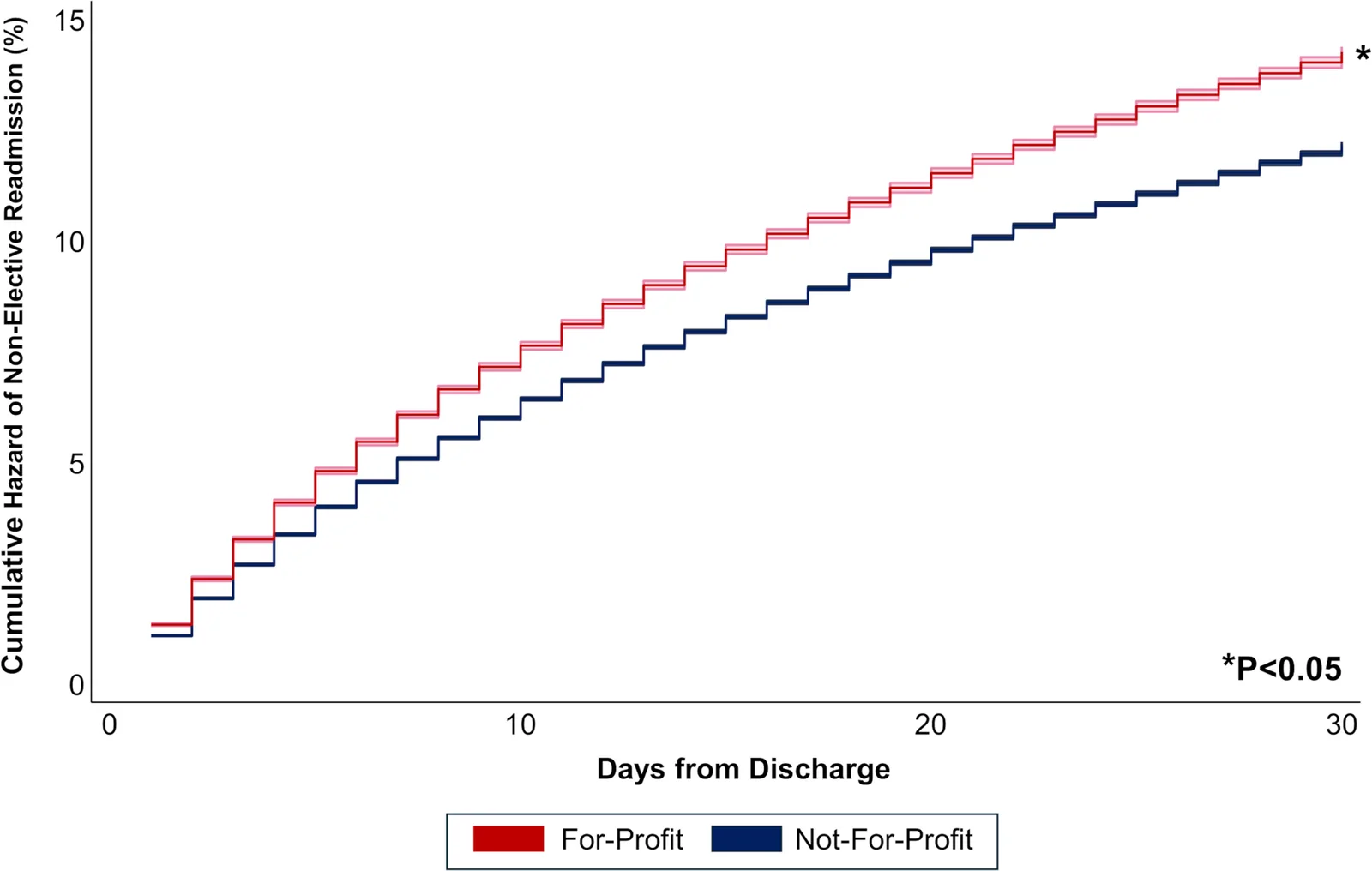
Cumulative Hazard of Readmission, Stratified by Hospital Ownership Cumulative hazards of 30-day nonelective readmission were examined using Nelson-Aalen methodology between *for-profit* and *not-for-profit* hospital cohorts. For-profit hospitals were associated with a higher cumulative hazard of 30-day nonelective readmission, compared with not-for-profit hospitals, suggesting a greater accumulated burden of readmission events over time. ∗Indicates statistical significance, *P* < 0.05. Shaded bands around each cumulative-hazard curve represent the 95% CIs.

**Figure 5:**
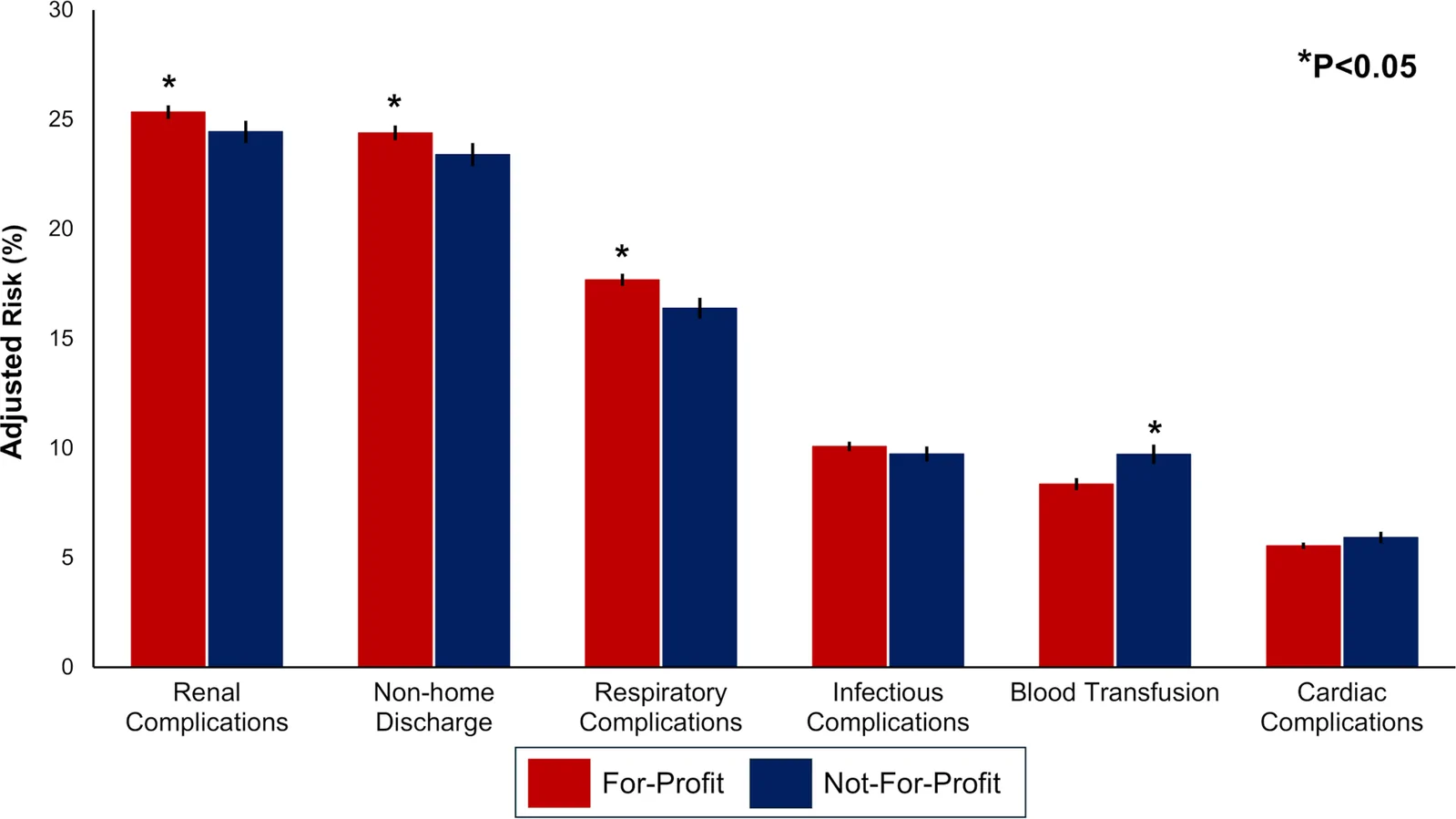
Risk-Adjusted Outcomes at Acute Myocardial Infarction Readmission by Hospital Ownership Following risk-adjustment and entropy balancing, for-profit hospitals remained associated with greater odds of nonhome discharge and renal and respiratory complications at readmission. Not-for-profit hospitals were associated with greater odds of blood transfusion during readmission. ∗Statistical significance, *P* < 0.05. Error bars represent the 95% CIs of the adjusted risk estimates.

The cumulative hazard of nonelective readmission was higher at for-profit hospitals than at not-for-profit hospitals throughout the 30 days after discharge (Figure 4). Following doubly robust risk adjustment, with not-for-profit as the reference group, *for-profit* was associated with similar odds of in-hospital mortality at readmission (AOR: 0.97; 95% CI: 0.91-1.02). *For-profit* was associated with decreased likelihood of cardiac complications (AOR: 0.92; 95% CI: 0.87-0.98) and blood transfusions (AOR: 0.85; 95% CI: 0.80-0.91). However, *for-profit* was linked with increased likelihood of respiratory (AOR: 1.11; 95% CI: 1.07-1.16) and renal complications (AOR: 1.04; 95% CI: 1.01-1.08). In addition, for-profit status was associated with a $4,298 decrease in hospitalization expenditures at index admission (95% CI: –$4,691 to –$3,906) and $2,753 decrease at readmission (95% CI: –$3,021 to –$2,485). Conversely, for-profit status corresponded with a 0.10-day increment in length of stay (95% CI: 0.01-0.19) and increased likelihood of nonhome discharge (AOR: 1.07; 95% CI: 1.03-1.11) (Table 3). Risk-adjusted outcomes for readmitted patients are shown on Figure 5. In the full index cohort, for-profit status was associated with greater adjusted odds of 30-day nonelective readmission compared with not-for-profit hospitals (AOR: 1.15; 95% CI: 1.14-1.17, *P* < 0.001) (Table 3). A Fine-Gray subdistribution hazard analysis treating in-hospital death at readmission as the competing event yielded concordant results (subdistribution HR: 1.18; 95% CI: 1.16-1.19; *P* < 0.001) (Supplemental Figure 1), suggesting the primary readmission finding is not explained by differential in-hospital mortality at readmission.

**Table 3:** Risk-Adjusted Outcomes of AMI Patients With a 30-Day Nonelective Readmission, Stratified by For-Profit Status

	Estimate (AOR/β)	95% CI
Primary outcome		
30-day nonelective readmission^a^	1.15	1.14-1.17
Clinical outcomes		
Mortality during readmission	0.97	0.91-1.02
Complications during readmission		
Cardiac	0.92	0.87-0.98
Respiratory	1.11	1.07-1.16
Infectious	1.04	0.99-1.09
Renal	1.04	1.01-1.08
Blood transfusion	0.85	0.80-0.91
Any	1.03	0.99-1.06
Resource utilization		
Hospitalization cost ($)	−4,298	−4,691, −3,906
Readmission costs ($)	−2,753	−3,021, −2,485
Length of stay (days)	+0.10	0.01-0.19
Nonhome discharge	1.07	1.03-1.11

The primary outcome, 30-day nonelective readmission, reflects the full index cohort (n = 4,636,970). Clinical and resource utilization outcomes are among patients readmitted to the index hospital (n = 399,800). Estimates from dichotomous outcomes were reported as AORs and continuous outcomes as β-coefficients; AOR, adjusted OR.

a Primary outcome reflects the full index admission cohort (n = 4,636,970); all other rows from readmitted cohort (n = 399,800).

## Discussion

In this national analysis of approximately 4.6 million AMI admissions, readmission rates declined at not-for-profit hospitals across all operative procedures and payer types from 2013 to 2022. Reductions at for-profit hospitals were observed only among CABG patients and Medicare beneficiaries. Rates of readmission remained higher at for-profit hospitals than at not-for-profit hospitals over the study period. Although risk of blood transfusion at readmission was lower, for-profit hospitals had higher risk of renal and respiratory complications at readmission than at not-for-profit hospitals. For-profit hospitals were associated with higher risk of nonhome discharge and a persistently higher cumulative hazard of nonelective readmission compared to not-for-profit hospitals (Central Illustration).

**Central Illustration:**
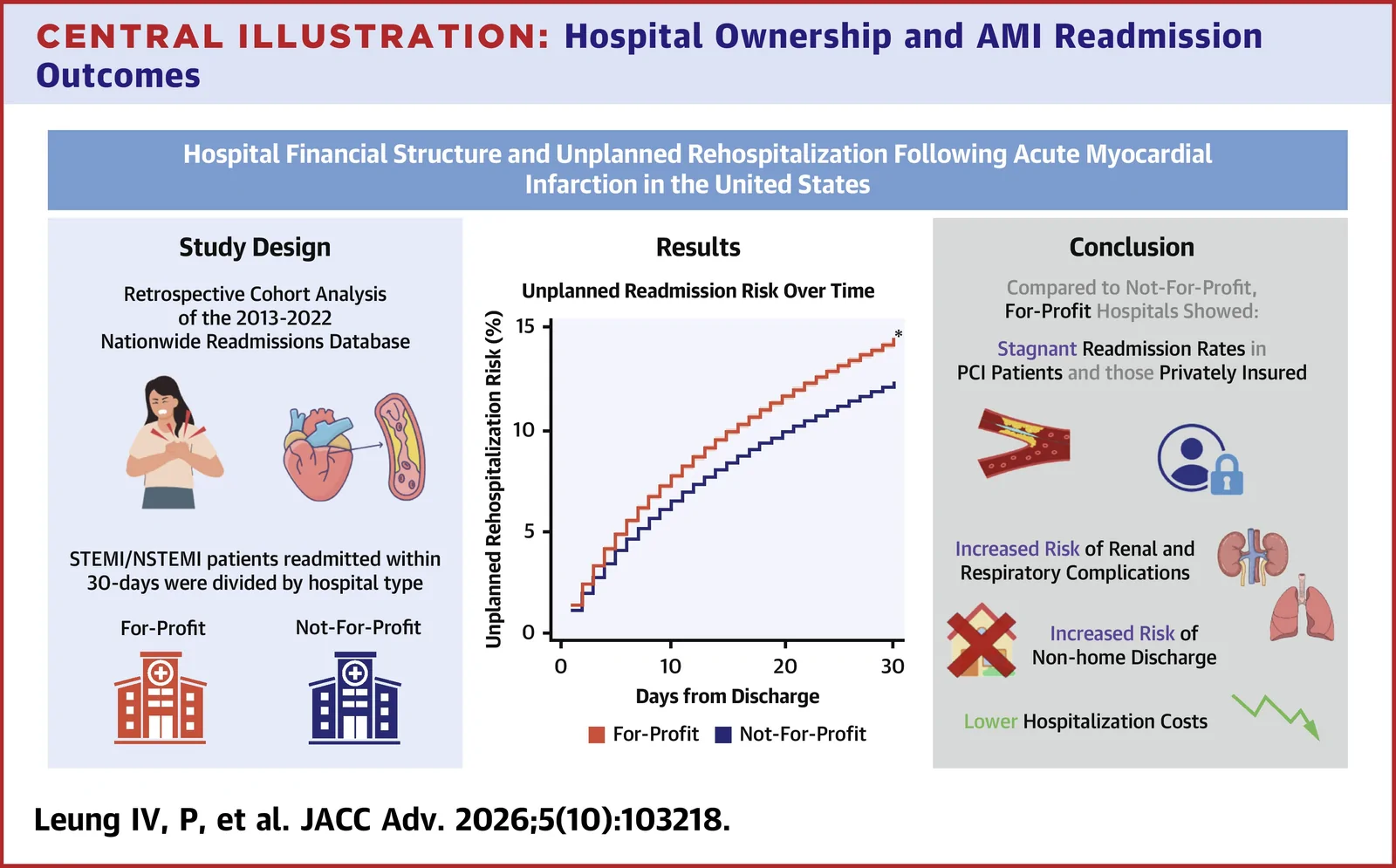
Hospital Ownership and AMI Readmission Outcomes Among 4,636,970 adults hospitalized for acute myocardial infarction in the United States from 2013 to 2022, for-profit hospitals were associated with persistently higher 30-day nonelective readmission rates compared with not-for-profit hospitals. Readmission rates declined across all procedures and payer types at not-for-profit hospitals over the study period, whereas reductions at for-profit hospitals were limited to coronary artery bypass grafting patients and Medicare beneficiaries. Following doubly robust risk adjustment, for-profit status was associated with greater odds of respiratory and renal complications at readmission and nonhome discharge. These findings identify ownership-related differences in AMI readmission outcomes with potential relevance to value-based care policy. NSTEMI = non–ST-segment elevation myocardial infarction; PCI = percutaneous coronary intervention; STEMI = ST-segment elevation myocardial infarction.

Not-for-profit hospitals showed significant reductions in readmission after both PCI and CABG, whereas for-profit hospitals showed a significant decline only in CABG readmissions (Figure 2). The observed difference in CABG vs PCI readmission trends at for-profit hospitals may relate to the higher baseline readmission rate and greater financial burden associated with CABG. CABG carries higher upfront procedural costs and longer lengths of stay, which may amplify the financial consequences of readmission for this procedure type.^20^ Reducing PCI-related readmissions may require outpatient infrastructure investments that differ across hospital ownership types.^10^^,^^21^ The absence of an overall decline at for-profit hospitals may in part reflect a dilution effect, where reductions in CABG-related readmissions were offset by unchanged rates among PCI patients. Observed reductions in readmission at not-for-profit hospitals are consistent with prior literature attributing improvements to structured reduction programs, revascularization during the index admission, and wider use of high-sensitivity troponin assays.^22^, ^23^, ^24^

AMI patients with Medicare and private insurance at not-for-profit hospitals showed significant decreases in 30-day nonelective readmission rates over the study period, whereas for-profit hospitals showed a notable decrease only among Medicare beneficiaries (Figure 3). These patterns are consistent with a differential HRRP response across hospital ownership types in the United States.^25^ Because HRRP created penalties specifically for Medicare fee-for-service beneficiaries, for-profit hospitals faced the strongest financial incentive to reduce readmissions in this population.^26^^,^^27^ Readmissions among privately insured patients do not incur HRRP penalties, which may attenuate the financial incentive to reduce readmissions in this group.^27^^,^^28^ The broader reduction in readmission rates observed at not-for-profit hospitals across all insurance categories may reflect differences in institutional priorities or organizational infrastructure.^29^^,^^30^

Compared with not-for-profit hospitals, for-profit status was associated with higher cumulative rehospitalization hazard (Figure 4), lower transfusion risk, and higher risks of renal and respiratory complications and nonhome discharge (Figure 5). These findings are consistent with prior studies, which found that for-profit hospitals were more likely to receive larger cumulative HRRP penalties and had higher readmission rates across major HRRP conditions compared to not-for-profit hospitals.^10^^,^^31^ One possible explanation is a difference in admission thresholds between ownership types. For-profit hospitals may admit lower-acuity patients at higher rates, exposing them to diagnostic and therapeutic work-up that may be deferred at not-for-profit hospitals, thereby accumulating complication risk.^32^ Differential coding across ownership types represents an additional alternative explanation that the present study cannot exclude. The lower risk of blood transfusion at for-profit hospitals may reflect differences in patient acuity at readmission.^33^ Higher nonhome discharge rates among for-profit patients may reflect ownership-associated differences in nursing capacity or discharge planning, consistent with prior work.^34^ Discharge planning and early outpatient follow-up may warrant particular attention at for-profit facilities.

### Study Limitations

As a retrospective administrative study, causal inference is precluded and findings are subject to selection bias and ICD-based coding variation. The NRD lacks granular clinical detail, limiting assessment of physiologic, laboratory, imaging, or procedural data. The NRD does not capture out-of-hospital events, outpatient procedures, or readmissions to hospitals in a different state, which may underestimate true 30-day readmission rates. Given that the NRD tracks patients within a single calendar year, long-term outcomes cannot be assessed. Hospital ownership in the NRD reflects tax and legal designation rather than operational practices, and behavioral differences between ownership types cannot be directly measured. E-values were calculated in the readmitted cohort to quantify the potential impact of unmeasured confounding. An unmeasured confounder would need a relative risk of at least 1.53 with both for-profit status and 30-day readmission to fully explain the primary association (lower confidence bound: 1.48). E-values for secondary outcomes, including renal complications at readmission, ranged from 1.20 to 1.66 (Supplemental Table 2). E-value analyses for risk-adjusted complications at the index admission, derived from models run in the full index cohort, are reported in Supplemental Table 2. An important limitation concerns out-of-hospital mortality. Because the NRD records only in-hospital death, deaths occurring after discharge are not observed. As underscored by the controversy over readmission-based quality metrics in heart failure, patients who die at home cannot be readmitted. Readmission and postdischarge mortality are therefore competing outcomes, and at least a substantial share of postevent deaths occur outside the hospital.^35^ If postdischarge survival differed by hospital ownership, the observed readmission comparison could be affected, and this possibility cannot be excluded in these data. These findings are therefore hypothesis-generating. The data that are available offer partial reassurance. In-hospital mortality was not higher at for-profit hospitals, either at the index admission (6.26% vs 6.53%) or, after risk adjustment, at readmission (AOR: 0.97). A Fine-Gray analysis treating in-hospital death at readmission as a competing event was also concordant with the primary result (Supplemental Figure 1). These analyses address only the in-hospital component of mortality. The out-of-hospital component remains unmeasured, and confirmation will require data that link readmission to postdischarge survival.

This national retrospective analysis identified persistent ownership-related differences in 30-day nonelective readmission rates and clinical outcomes following AMI. Not-for-profit hospitals demonstrated significant reductions in readmission rates across operative procedures and payer types over the study period. For-profit hospitals showed significant declines in readmission rates among CABG patients and Medicare beneficiaries, a pattern consistent with the payer-specific scope of HRRP financial penalties. For-profit status was associated with decreased risk of blood transfusion and increased risk of respiratory and renal complications at readmission, along with higher rates of nonhome discharge. Temporal trend analysis indicated a persistently higher rate of 30-day nonelective readmission at for-profit hospitals compared with not-for-profit hospitals. These observations may be relevant to policy efforts targeting ownership-related disparities in AMI readmissions.

Perspectives **COMPETENCY IN SYSTEMS-BASED PRACTICE:** For-profit hospital status is associated with higher 30-day nonelective readmission rates and greater odds of renal and respiratory complications and nonhome discharge following AMI. Privately insured patients at for-profit centers showed no reduction in readmission from 2013 to 2022, in contrast to declines across insurance categories at not-for-profit hospitals. Discharge planning and early outpatient follow-up warrant particular attention at for-profit facilities.

**TRANSLATIONAL OUTLOOK:** Future research should determine whether discharge planning infrastructure and postdischarge care coordination explain ownership-associated variation in AMI readmission outcomes, and whether standardized care coordination protocols reduce ownership-based disparities. The differential response to the HRRP by payer type warrants consideration in policy design targeting AMI readmissions across all insurance types.

## Funding support and author disclosures

Dr Benharash has received proctoring fees from AtriCure. All other authors have reported that they have no relationships relevant to the contents of this paper to disclose.
