# Supplementary material for: Hospital Financial Structure and Unplanned Rehospitalization Following Acute Myocardial Infarction in the United States
Source: JACC Adv. 2026 Sep 10;5(10):103218. doi: 10.1016/j.jacadv.2026.103218 (PMC13586794; doi:10.1016/j.jacadv.2026.103218)
Supplement: Supplementary file 1 [file mmc1.pdf]

Supplemental Table S1. Diagnosis and Procedure Codes. *ICD-10*, International Classification of Diseases, Tenth Revision diagnosis and procedure codes; *ICD-9*, International Classification of Diseases, Ninth Revision diagnosis and procedure codes.

Diagnosis / Procedure	ICD-10 Codes	ICD-9 Codes
Acute Myocardial Infarction (AMI)		
Non-ST-elevation myocardial infarction (NSTEMI)	I21.4, I22.2	410.71
ST-elevation myocardial infarction (STEMI)[a]	I21.01, I21.02, I21.09, I21.11, I21.19, I21.21, I21.29, I21.3, I21.9	410.01, 410.11, 410.21, 410.31, 410.41, 410.51, 410.61, 410.81, 410.91
Operative Procedures		
Coronary Artery Bypass Grafting (CABG)	02100XX[b], 02110XX[b], 02120XX[b], 02130XX[b]	36.10, 36.11, 36.12, 36.13, 36.14, 36.15, 36.16, 36.17, 36.19
Percutaneous Coronary Intervention (PCI)	0270346, 027034Z, 02703D6, 02703DZ, 02703Z6, 02703ZZ, 0270446, 027044Z, 02704D6, 02704DZ, 02704Z6, 02704ZZ, 0271346, 027134Z, 02713D6, 02713DZ, 02713Z6, 02713ZZ, 0271446, 027144Z, 02714D6, 02714DZ, 02714Z6, 02714ZZ, 0272346, 027234Z, 02723D6, 02723DZ, 02723Z6, 02723ZZ, 0272446, 027244Z, 02724D6, 02724DZ, 02724Z6, 02724ZZ, 0273346, 027334Z, 02733D6, 02733DZ, 02733Z6, 02733ZZ, 0273446, 027344Z, 02734D6, 02734DZ, 02734Z6, 02734ZZ	00.66, 36.06, 36.07

Diagnosis / Procedure	ICD-10 Codes	ICD-9 Codes
Cardiac Complications		
Cardiac arrest	I46.2, I46.8, I46.9	427.5
Ventricular tachycardia	I47.2	427.1
Ventricular fibrillation	I49.01	427.41
Cardiac tamponade	I31.4	423.3
Respiratory Complications		
Pneumonia	J13, J14, J12.0, J12.1, J12.2, J12.9, J12.81, J12.89, J15.0, J15.1, J15.3, J15.4, J15.5, J15.6, J15.7, J15.8, J15.9, J15.20, J15.29, J15.211, J15.212, J16.0, J16.8, J18.0, J18.1, J18.9, J95.851, J95.89	480.0-480.3, 480.8-480.9, 482.0-482.2, 482.9, 483.0-483.1, 483.8, 482.30-482.32, 482.39-482.49, 482.81-482.84, 482.89, 481, 485, 486, 997.31, 997.32
Pneumothorax	J95.811	512.1
Acute respiratory distress syndrome (ARDS)	J80, R06.03	518.5, 518.82
Respiratory failure	J96.00, J96.20, J96.90, J95.821, J95.822	518.81, 518.51, 518.53, 518.84
Prolonged mechanical ventilation	5A1955Z	96.72
Bronchopleural fistula	J86.0	510.0

Infectious Complications

Sepsis	A40.3, A40.9, A41.01, A41.02, A41.1, A41.2, A41.3, A41.4, A41.50, A41.51, A41.52, A41.53, A41.59, A41.89, A41.9, R65.20, T80.211A, T80.212A, T80.219A, T80.22XA, T80.29XA, T81.4XXA, K68.11	038, 038.1-038.19, 038.2, 038.3, 038.4-038.49, 038.8, 038.9, 995.91, 995.92, 999.3
Surgical site infection	T81.31XA, T81.32XA, T81.4XXA[c], K68.11	998.31, 998.32, 998.5

Renal Complications

Acute kidney injury (AKI)[d]	N17.0, N17.1, N17.2, N17.8, N17.9	584, 584.5-584.9

Blood Transfusion *(not included in Any Complication composite)*

Blood transfusion	30233H0, 30233N0, 30233W0, 30243H0, 30243N0, 30243W0, 30253H0, 30253N0, 30253W0, 30263H0, 30263N0, 30263W0, 30233H1, 30243H1, 30253H1, 30263H1, 30233N1-30263N1, 30233P1-30263P1, 3E033GC, 3E043GC, 3E053GC, 3E063GC	990.xx

<u>Additional Components of Any Complication Composite</u>

Thromboembolic Complications

Deep vein thrombosis	I80.10, I80.209, I80.3, I80.219, I80.9, I82.220, I82.409, I82.419, I82.429, I82.439, I82.449, I82.499, I82.4Y9, I82.4Z9, I82.619, I82.629, I82.609, I82.A19, I82.B19, I82.C19, I82.290, I82.890, I82.91	451.1, 451.2, 451.81, 451.9, 453.2, 453.40-453.42, 453.8, 453.9
Pulmonary embolism	I26.90, I26.99, T80.0XXA, T81.718A, T81.72XA, T82.817A, T82.818A	415.1, 415.11, 415.12, 415.19
Stroke		
Intracranial hemorrhage	I60.9, I61.9, I62.0, I62.1, I62.9	430, 431, 432.0-432.9
Acute ischemic stroke	I63.019, I63.119, I63.139, I63.219, I63.239, I63.20, I63.30, I63.40, I63.50, I63.59, I67.2, I67.5, I67.7, I67.81, I67.82, I67.89, I67.9, G45.4	433.01, 433.11, 433.21, 433.31, 433.81, 433.91, 434.01, 434.11, 434.91, 437.0, 437.1, 437.4, 437.5, 437.7, 437.9
Perioperative stroke	G97.81, G97.82, I97.811, I97.821	997.01, 997.02
Intraoperative Complications		
Accidental puncture / laceration	D78.11-D78.12, E36.11-E36.12, G97.48-G97.49, H59.219, H59.229, H95.31-H95.32, I97.51-I97.52, J95.71-J95.72, K91.71-K91.72, L76.11-L76.12, M96.820-M96.821, N99.71-N99.72, T88.8XXA	998.2

Procedural hemorrhage	D78.01-D78.02, D78.21-D78.22, E36.01-E36.02, G97.31-G97.32, G97.51-G97.52, H59.111-H59.129, H59.311-H59.329, H95.21-H95.22, I97.410-I97.411, I97.42, I97.610-I97.611, J95.61-J95.62, J95.830-J95.831, K91.61-K91.62, L76.01-L76.02, L76.21-L76.22, M96.810-M96.811, N99.61-N99.62, N99.820-N99.821	998.11
Phrenic nerve injury	N/A[e]	354.8, 519.4
Vagus nerve injury	N/A[e]	951.8

[a]Subsequent myocardial codes (I22.0, I22.1, I22.8, I22.9) were excluded from the STEMI definition. These codes denote subsequent acute myocardial infarctions occurring within 4 weeks of an index MI and were excluded to avoid double-counting of prevalent AMI hospitalizations.

[b]XX indicates all possible character variations.

[c]T81.4XXA is captured in both the sepsis and surgical site infection definitions but does not affect the composite.

[d]AKI defined excluding patients with pre-existing end-stage renal disease or chronic dialysis dependence at the index admission.

[e]No ICD-10-CM code of comparable specificity exists for this complication. Cases were captured only in ICD-9-CM coded discharges (2013 to 2015).

Supplemental Table S2: E-Value Analysis for Primary Adjusted Outcomes. E-values were calculated using the VanderWeele and Ding method. Adjusted odds ratios were converted to approximate risk ratios using baseline outcome risks (p_0) from the not-for-profit reference group. An E-value is the minimum risk ratio an unmeasured confounder would require with both for-profit status and the outcome to fully explain the observed association. Readmission and readmission outcome analyses were performed in the readmitted cohort (N=399,800). Index admission complication analyses were performed in the full index cohort. AOR, adjusted odds ratio; CI, confidence interval; RR, risk ratio; AKI, acute kidney injury.

Outcome	AOR (95% CI)	Baseline Risk (p_0)	Approx RR	E-value	E-value (lower CI)
30-Day Non-Elective Readmission					
30-day readmission (entropy-balanced)	1.15 (1.14-1.17)	0.11	1.13	1.53	1.48
30-day readmission (unadjusted)	1.16 (1.14-1.18)	0.11	1.14	1.54	1.50
Complications at Readmission					
Respiratory complications	1.11 (1.07-1.16)	0.15	1.09	1.41	1.30
Renal complications (AKI)	1.04 (1.01-1.08)	0.25	1.03	1.20	1.09
Non-home discharge	1.07 (1.03-1.11)	0.23	1.05	1.29	1.18
Complications at Index Admission					
Respiratory complications	1.23 (1.19-1.26)	0.15	1.19	1.66	1.59
Renal complications (AKI)	1.21 (1.18-1.24)	0.25	1.15	1.56	1.51
Non-home discharge	1.05 (1.03-1.08)	0.18	1.04	1.25	1.17

Supplemental Figure S1: Competing Risk Analysis Plot. Cumulative incidence of 30-day non-elective readmission estimated using the Fine-Gray subdistribution hazard model, with in-hospital death at readmission as the competing event. For-profit hospitals demonstrated persistently greater cumulative incidence across the full 30-day window, consistent with the primary Nelson-Aalen finding (Figure 4). Subdistribution hazard ratio for for-profit versus not-for-profit is 1.18 (95% CI 1.16-1.19, P<0.001). These results suggest the readmission difference between ownership types is not attributable to differential in-hospital mortality. * indicates statistical significance, P<0.05. *FP*, for-profit; *NFP*, not-for-profit; *CI*, confidence interval.

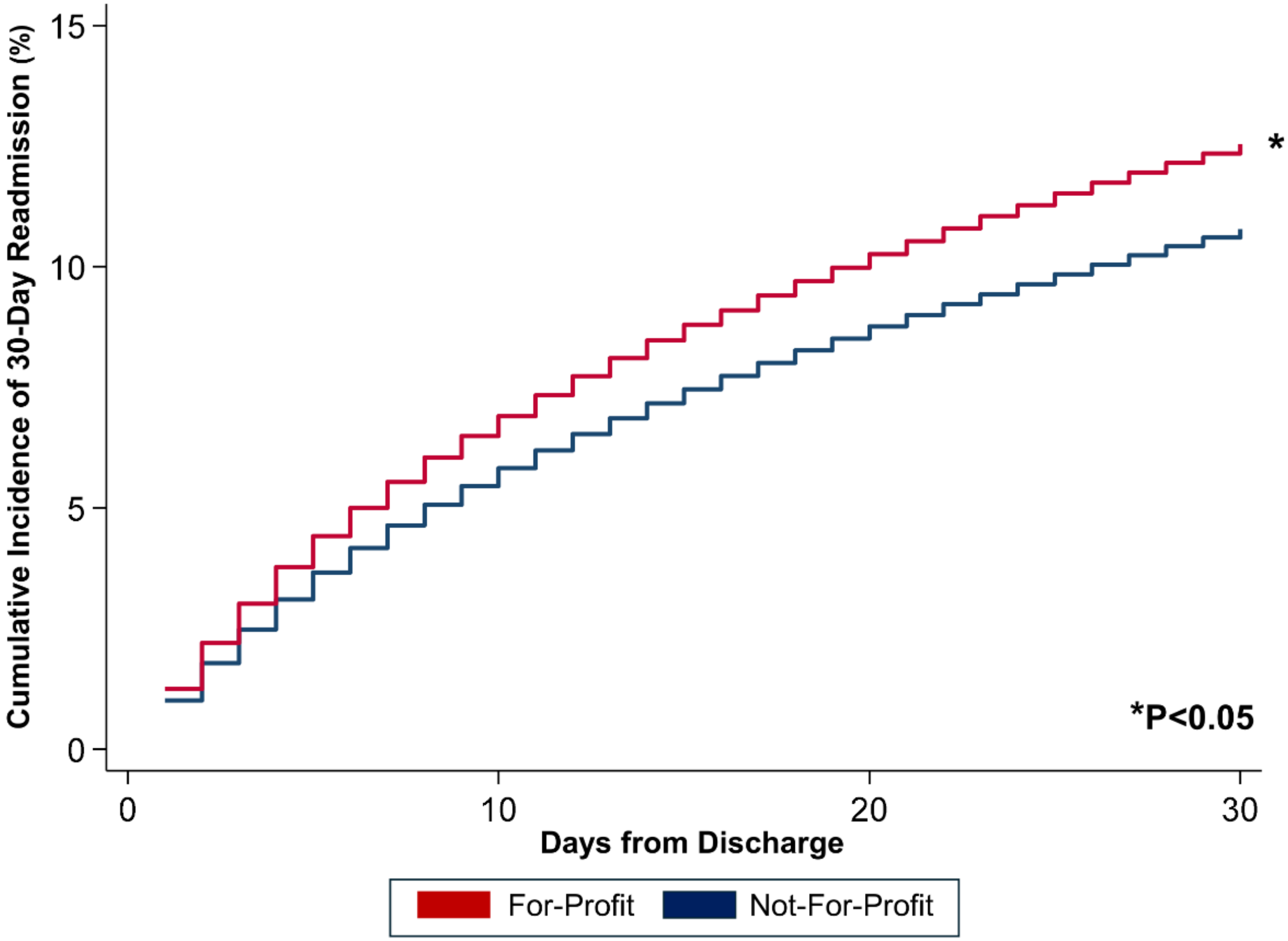

STROBE Statement—checklist of items that should be included in reports of observational studies

	Item No.	Recommendation	Page No.	Relevant text from manuscript
Title and abstract	1	(a) Indicate the study's design with a commonly used term in the title or the abstract	1	Title: Hospital Financial Structure and Unplanned Rehospitalization Following Acute Myocardial Infarction in the United States
		(b) Provide in the abstract an informative and balanced summary of what was done and what was found	2-3	Abstract
Introduction				
Background/rationale	2	Explain the scientific background and rationale for the investigation being reported	6-7	Introduction – Background
Objectives	3	State specific objectives, including any prespecified hypotheses	6-7	Introduction – Hypothesis
Methods				
Study design	4	Present key elements of study design early in the paper	7-9	Methods – Retrospective study
Setting	5	Describe the setting, locations, and relevant dates, including periods of recruitment, exposure, follow-up, and data collection	7-9	Methods – 2013-2022
Participants	6	(a) *Cohort study*—Give the eligibility criteria, and the sources and methods of selection of participants. Describe methods of follow-up<br>*Case-control study*—Give the eligibility criteria, and the sources and methods of case ascertainment and control selection. Give the rationale for the choice of cases and controls<br>*Cross-sectional study*—Give the eligibility criteria, and the sources and methods of selection of participants	7-9	Methods – All adult (≥18 years) non-elective hospitalizations for AMI
		(b) *Cohort study*—For matched studies, give matching criteria and number of exposed and unexposed<br>*Case-control study*—For matched studies, give matching criteria and the number of controls per case		
Variables	7	Clearly define all outcomes, exposures, predictors, potential confounders, and effect modifiers. Give diagnostic criteria, if applicable	7-9	Methods – Characteristics

Data sources/ measurement	8*	For each variable of interest, give sources of data and details of methods of assessment (measurement). Describe comparability of assessment methods if there is more than one group	7-9	Methods – Variables
Bias	9	Describe any efforts to address potential sources of bias	14-15	Discussion – Limitations
Study size	10	Explain how the study size was arrived at	7-9	Methods

Continued on next page

Quantitative variables	11	Explain how quantitative variables were handled in the analyses. If applicable, describe which groupings were chosen and why	7-9	Methods – Characteristics
Statistical methods	12	(a) Describe all statistical methods, including those used to control for confounding	7-9	Statistical Methods
		(b) Describe any methods used to examine subgroups and interactions	NA	NA
		(c) Explain how missing data were addressed	7-9	Methods
		(d) *Cohort study*—If applicable, explain how loss to follow-up was addressed<br>*Case-control study*—If applicable, explain how matching of cases and controls was addressed<br>*Cross-sectional study*—If applicable, describe analytical methods taking account of sampling strategy	NA	NA
		(e) Describe any sensitivity analyses	12, 14-15	Results – Supplemental Figure S1; Supplemental Table S2
Results				
Participants	13*	(a) Report numbers of individuals at each stage of study—eg numbers potentially eligible, examined for eligibility, confirmed eligible, included in the study, completing follow-up, and analysed	9-12	Results
		(b) Give reasons for non-participation at each stage	NA	NA
		(c) Consider use of a flow diagram	21	Figure 1
Descriptive data	14*	(a) Give characteristics of study participants (eg demographic, clinical, social) and information on exposures and potential confounders	24	Table 1
		(b) Indicate number of participants with missing data for each variable of interest	21	Figure 1
		(c) *Cohort study*—Summarise follow-up time (eg, average and total amount)	NA	NA
Outcome data	15*	*Cohort study*—Report numbers of outcome events or summary measures over time	9-12	Results
		Case-control study—Report numbers in each exposure category, or summary measures of exposure	NA	NA
		Cross-sectional study—Report numbers of outcome events or summary measures	NA	NA
Main results	16	(a) Give unadjusted estimates and, if applicable, confounder-adjusted estimates and their precision (eg, 95% confidence interval). Make clear which confounders were adjusted for and why they were included	9-12	Results
		(b) Report category boundaries when continuous variables were categorized	9-12	Results
		(c) If relevant, consider translating estimates of relative risk into absolute risk for a meaningful time period	22	Figure 5

Continued on next page

Other analyses	17	Report other analyses done—eg analyses of subgroups and interactions, and sensitivity analyses	9-12	Results
Discussion				
Key results	18	Summarise key results with reference to study objectives	12-15	Discussion
Limitations	19	Discuss limitations of the study, taking into account sources of potential bias or imprecision. Discuss both direction and magnitude of any potential bias	14-15	Limitations
Interpretation	20	Give a cautious overall interpretation of results considering objectives, limitations, multiplicity of analyses, results from similar studies, and other relevant evidence	12-15	Discussion
Generalisability	21	Discuss the generalisability (external validity) of the study results	12-15	Discussion
Other information				
Funding	22	Give the source of funding and the role of the funders for the present study and, if applicable, for the original study on which the present article is based	NA	NA

*Give information separately for cases and controls in case-control studies and, if applicable, for exposed and unexposed groups in cohort and cross-sectional studies.

Note: An Explanation and Elaboration article discusses each checklist item and gives methodological background and published examples of transparent reporting. The STROBE checklist is best used in conjunction with this article (freely available on the Web sites of PLoS Medicine at http://www.plosmedicine.org/, Annals of Internal Medicine at http://www.annals.org/, and Epidemiology at http://www.epidem.com/). Information on the STROBE Initiative is available at www.strobe-statement.org.
